# A cross‐sectional study of stool form (using Bristol stool chart) in an urban South Indian population

**DOI:** 10.1002/jgh3.12189

**Published:** 2019-04-25

**Authors:** Melpakkam Srinivas, Vijaya Srinivasan, Mayank Jain, Coimbatore Subramanian Rani Shanthi, Viswanathan Mohan, Viswanathan Jayanthi

**Affiliations:** ^1^ Department of Gastroenterology Gleneagles Global Health City Chennai India; ^2^ Dr. Mohan's Diabetes Specialities Centre and Madras Diabetes Research Foundation Chennai India

**Keywords:** Bristol stool form, healthy, South India, urban

## Abstract

**Background:**

There is paucity of data on Bristol stool form (BSF) in healthy South Indian residents.

**Aim:**

To determine the BSF types and associated factors in an urban bowel health noncomplainant population.

**Methods:**

This cross‐sectional study, performed using a self‐administered questionnaire among adult Chennai residents, compared BSF types by gender for various factors (age, occupation, bowel frequency, and defecation‐related abdominal pain). BSF types 1/2 and 6/7 were grouped as hard and loose stools, respectively. The statistical tests used were proportion test, *χ* ^2^, and Kruskal–Wallis tests (*P* < 0.05 deemed significant).

**Results:**

The study cohort of 1402 subjects included 748 (53.3%) men and a third each of professionals, semiprofessionals, and “non‐office goers” (homemakers, retirees, students, and unemployed). A total of 97% had daily bowel movement, and 8.5% reported defecation associated abdominal pain. The BSF types in decreasing prevalence were: Type 3 (35.6%), Type 4 (32.5%), Types 1 or 2 (20.5%), Type 5 (6.9%), and Types 6 or 7 (4.5%). On gender comparison, significantly more men passed hard (*P* = 0.03) or loose stools (*P* = 0.001), while more women passed Type 3 (*P* = 0.0002). Loose stools in men were associated with abdominal pain (*P* = 0.0035). Women passing hard or loose stool types were slightly older (median age in 30s vs. 20s in Types 3–5) and had reduced stool frequency (*P* = 0.026: hard; *P* = 0.006: loose).

**Conclusions:**

This South Indian noncomplainant cohort's most common stool types were BSF Types 3 and 4, with few gender variations in extreme stool types.

## Graphical abstract

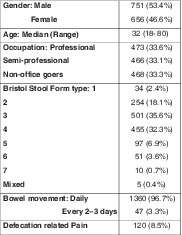

This first study of stool form in south Indian noncomplainants shows that the majority pass Bristol stool Types 3 and 4. There are few gender variations only in the extremes of stool types.

## Introduction

Stool frequency and form are important parameters that characterize normal bowel pattern in a given population. These serve as the baseline to delineate stool patterns in various disease states. In the Western population, stool frequency of 3–21 per week is considered normal.^1^, ^2^ However, studies on Asian populations have reported higher mean stool frequency.^3^, ^4^ There is only one study^4^ from India classifying stool form using the Bristol stool form scale (BSF) in the general population.

The present population‐based, cross‐sectional study aims to report the stool type using BSF and the factors associated with them in adult residents of Chennai city (southern India) with no bowel‐related complaints (noncomplainants).

## Materials and methods

This cross‐sectional survey of a purposive sample of adults (aged more than 17 years) elicited information on basic demographics, stool patterns, and frequency. The sampling was performed on a simple random selection of people who encountered the researchers at health awareness talk sessions in government and private organizations, as well as apparently healthy relatives accompanying outpatients to two large corporate hospitals. Those in employment were classified according to the International Standard Classification of Occupations (ISCO) 2008^5^ as professional and semiprofessional.

After obtaining informed verbal consent, data were collected by self‐administration of a formatted, standardized, pretested questionnaire, ensuring 95% coverage of the sampled population. The questionnaire collected basic demographic data (gender, age, occupation, and period of residence in Chennai) and medical information on previously diagnosed bowel diseases such as intestinal tuberculosis, inflammatory bowel disease (IBD), fissures, and hemorrhoids. Data on bowel pattern, including BSF type, frequency, presence of blood or mucus in stool, and defecation‐related abdominal pain were provided by each volunteer only once based on a 2‐week recall of their “normal” bowel habits.

Those reporting blood or mucus in stool, other bowel disorders like IBD and anal fissure, or those on long‐term medications that could interfere with bowel movement, for example, antipsychotic drugs, were excluded from this study. Thus, the study population included only those with no bowel‐related complaints (i.e. bowel health noncomplainants).

Based on the concordant stool forms of Rome IV^6^ and Asian^7^ classifications, BSF Types 1 and 2 were combined as definitely hard, and Types 6 and 7 were combined as definitely loose. Types 3, 4, and 5 were reported as individual groups. Subjects reporting both hard and loose BSF types were categorized as “mixed” type. The BSF types were then compared by gender. If significant differences were found between genders, the remaining factors (age, occupation, stool frequency, and defecation‐associated pain) were analyzed by BSF type for each gender.

### *Statistical tests*

Data were analyzed using SPSS version 20 software. Categorical variables were reported as percentages and continuous variables not normally distributed as median and range. Proportions were compared using the proportion test and *χ* ^2^ test as appropriate. Medians were compared using the Kruskal–Wallis (KW) test. Factors found to be significant on univariate analysis were subjected to multivariate analysis. A *P* value < 0.05 was deemed significant for all the statistical tests applied.

Ethical approval for the survey was sanctioned by the Institutional Ethics Committee.

## Results

### *Baseline characteristics of the study population*

This predominantly young cohort (median age 32 years) of 1407 subjects consisted of a slightly higher proportion of males (53%) (proportion test, *P* = 0.011) and a similar proportion (a third each) of professionals (ISCO major group 2), semiprofessionals(ISCO major groups 3 and 4), and nonoffice goers (*P* = 0.082) (Table 1). The “nonoffice goers” comprised of homemakers, retirees, students, and the unemployed. All the subjects were residents of Chennai at the time of interview, with 96.9% being a resident for more than 1 year.

**Table 1:** Baseline characteristics of study population (*n* = 1407)

Gender	
Male	751 (53.4%)
Female	656 (46.6%)
Age	
Median (range)	32 (18–80)
Occupation	
Professional	473 (33.6%)
Semiprofessional	466 (33.1%)
Nonoffice goers	468 (33.3%)
Bristol stool form type	
1	34 (2.4%)
2	254 (18.1%)
3	501 (35.6%)
4	455 (32.3%)
5	97 (6.9%)
6	51 (3.6%)
7	10 (0.7%)
Mixed	5 (0.4%)
Bowel movement	
Daily	1360 (96.7%)
Every 2–3 days	47 (3.3%)
Defecation‐related Pain	120 (8.5%)

Almost all (96.7%) subjects passed motion daily, with the rest having bowel movements every 2 or 3 days. On the days of bowel movement, 97.6% passed 1–2 stools, and the rest reported ≥ 3 stools. Defecation‐related abdominal pain was reported by 8.5% of the cohort.

BSF Types 3 (35.5%) and 4 (32.4%) were the most common stool types, followed by Types 1 and 2 (hard stools) in 20.5%. BSF Type 5 and BSF Types 6 and 7 (loose stools) were relatively uncommon at 6.9% and 4.3%, respectively.

Only five subjects (0.4%) reported mixed type of stools, comprising three men, four professionals, with a median age of 26 years, a daily bowel movement in four and defecation‐related abdominal pain in three. This mixed stool category was not included for further analysis due to the very low prevalence.

### *Comparison of BSF types by gender*

The BSF types in the remaining study population of 1402 subjects were compared by gender (Table 2). Overall, irrespective of gender, BSF Types 3 and 4 were the most commonly reported stool types. However, a significantly higher proportion of men reported hard (22.7% vs. 18%; *P* = 0.03) and loose (6% vs. 2.5%; *P* = 0.001) stools compared to women. Significantly more women reported Type 3 stools than men (31.3% vs. 40.8%; *P* = 0.0002).

**Table 2:** Stool type by gender

	BSF type					
Gender	Hard: 1/2	3	4	5	Loose: 6/7	*P*
Male (748)	170 (22.7%)	234 (31.3%)	241 (32.2%)	58 (7.8%)	45 (6%)	0.00005
Female (654)	118 (18%)	267 (40.8%)	214 (32.7%)	39 (6%)	16 (2.5%)	

BSF, Bristol stool form.

In view of significant gender variations in BSF types, analysis of the remaining factors was performed separately for each gender.

### *Determination of factors associated with BSF types in men*

In men, defecation‐related abdominal pain with loose stool (BSF 6/7) was the only significant factor (17.6% with pain vs. 5.4% without pain; *P* = 0.0035) (Table 3). All the other factors, including age, occupation, and stool frequency, were similar irrespective of stool type. The median age across all stool types was between 32 and 36 years.

**Table 3:** Univariate analysis in men by Bristol stool form (BSF) type

	BSF type					
Factors	Hard: 1/2	3	4	5	Loose: 6/7	*P*
Age						
Median (range)†	36 (18–80)	36 (20–80)	32 (18–77)	34 (22–66)	32 (19–68)	0.0646
Occupation						
Professional (362)	77 (21.2%)	115 (31.8%)	113 (31.2%)	36 (9.9%)	21 (5.8%)	0.051
Semiprofessional (284)	70 (24.6%)	77 (27.1%)	104 (36.6%)	17 (6%)	16 (5.6%)	
Nonoffice goers (102)	23 (22.5%)	42 (41.2%)	24 (23.5%)	5 (4.9%)	8 (7.8%)	
Bowel movement						
Daily (732)	168 (30%)	229 (31.3%)	235 (32.1%)	56 (7.6%)	44 (6%)	0.85
Every 2–3 days (16)	2 (12.5%)	5 (31%)	6 (38%)	2 (12.5%)	1 (6%)	
Defecation related						
Pain (34)	11 (32.4%)	10 (29.4%)	6 (17.6%)	1 (2.9%)	6 (17.6%)	0.0117
No pain (714)	159 (22.3%)	224 (31.4%)	234 (32.8%)	58 (8.1%)	39 (5.4%)	

† Kruskal–Wallis test.

### *Determination of factors associated with BSF types in women*

In women, extremes of stool types (hard or loose) were significantly associated with higher median age than those with BSF Types 3, 4, or 5 (third vs. second decade) (Table 4). Similarly, extremes of stool types were significantly associated with reduced bowel movement frequency of every 2–3 days (hard: 17.3% vs. 33.3%; *P* = 0.026 and loose: 2.1% vs. 10%; *P* = 0.006), although only a small proportion of women passed loose stools (2.4%). There was no significant association between BSF types and occupation or defecation‐related abdominal pain.

**Table 4:** Univariate analysis in women by Bristol stool form (BSF) type

	BSF type					
Factors	Hard: 1/2	3	4	5	Loose: 6/7	*P*
Age						
Median (range)†	32 (20–72)	28 (19–80)	28 (19–75)	25 (19–59)	34.5 (19–68)	0.049
Occupation						
Professional (107)	22 (20.6%)	38 (35.5%)	35 (32.7%)	8 (7.5%)	4 (3.7%)	0.59
Semiprofessional (181)	27 (14.9%)	80 (44.2%)	57 (31.5%)	14 (7.7%)	3 (1.7%)	
Nonoffice goers (366)	69 (18.9%)	149 (40.7%)	122 (33.3%)	17 (4.6%)	9 (2.5%)	
Bowel movement						
Daily (624)	108 (17.3%)	257 (41.2%)	209 (33.5%)	37 (5.9%)	13 (2.1%)	0.006
Every 2–3 days (30)	10 (33.3%)	10 (33.3%)	5 (16.7%)	2 (6.7%)	3 (10%)	
Defecation related						
Pain (83)	23 (27.7%)	29 (34.9%)	24 (28.9%)	5 (6%)	2 (2.5%)	0.19
No pain (571)	95 (16.6%)	238 (41.7%)	190 (33.3%)	34 (6%)	14 (2.5%)	

† Kruskal–Wallis test.

## Discussion

This study from South India reporting stool frequency and form in a cross section of noncomplainant Chennai city residents shows that the most common stools types irrespective of gender were BSF Types 3 and 4, accounting for about two‐thirds of the study population. Almost all subjects had daily bowel movement, and defecation‐associated abdominal pain occurred in a minority (8.5%).

Our study showed significant gender variations associated with BSF types. Men passed extremes of stool types (hard and loose stools) more commonly than women. A higher proportion of women passed Type 3 stools compared to men. While BSF Types 3–5 (normal by Rome IV classification) were not significantly influenced by any of the other factors studied, both genders demonstrated distinct characteristics at extremes of stool types. Men had abdominal pain with loose stools. Median age of women passing hard and loose stools were higher compared to BSF Types 3–5 (third *vs* second decade), and a significantly higher proportion of them did not have daily bowel movement.

A small study from the United States^8^ on 32 healthy volunteers reported that women passed significantly hard stools, and stool form correlated with colon transit time (slower in those with hard stools) but not stool frequency. We found that women tended to pass harder stools (BSF Type 3) than men and had reduced stool frequency with extremes of stool types. While lesser frequency with hard stools is understandable, the same is counterintuitive with loose stools. Possible reasons include overflow diarrhea (actually constipated) and obstructive defecation; both of which have not been assessed in detail in our study.

A previous study on 271 residents of Bishan (Singapore)^9^ had reported daily bowel movement in only 59% respondents, in contrast to our population where almost 97% had daily bowel movement. The high proportion with daily bowel movement in our study is consistent with the previously reported whole gut transit time of 25.8 h amongst healthy controls from South India.^10^ Furthermore, the Indian Society of Gastroenterology task force on irritable bowel syndrome in India^11^ surveyed 4500 noncomplainants representing different regions of the country, predominantly from the lower socioeconomic strata, and reported that almost all (99%) the respondents had bowel frequency of at least once a day. However, BSF type was not reported in the study.

A single‐center study^12^ from eastern India (Kolkata) on 331 patients with complaints of constipation reported BSF Types 1 or 2 in 67.9% (20.5% in our study) and daily defecation in only 19.8% (cf. 96.8%). BSF 3, reported by 25.9% (cf. 35.6%), was significantly associated with normal stool frequency. As highlighted in parenthesis, our BSF type and stool frequency profile were different from this patient cohort, suggesting that our noncomplainant sample probably represents the healthy population.

A further key finding in that study was that the feeling of incomplete evacuation was universal and referred to as constipation by patients. This suggests that the mere passage of hard stools (BSF Types 1–3 in 56.1% of our noncomplainant sample) does not imply disease, and the perceptions of ill‐health such as incomplete evacuation are more relevant in functional disorders.

The only population‐based study^4^ till date from India on BSF type was performed on 1200 subjects from the eastern Indian state of Odisha. The authors reported an average stool frequency of 14 per week, with predominant stool form of BSF Type 4 (58.3%) followed by Type 6 (14.8%). On multivariate analysis, women and age > 35 years were associated with less stool frequency. In contrast to this study, we noted that 72% of our subjects opened their bowels only once a day, and the most common stool type was BSF 3, followed by Type 4. However, similar to this study, we also found that older women (beyond the second decade) had lesser bowel frequency with both hard and loose stools. Further comparisons between the two study populations to determine reasons for the above variations is not possible as the socioeconomic status of the Odisha cohort was not reported, and we did not collect dietary and physical activity details in our volunteers. However, the differences in stool form and bowel movement pattern between these two populations suggest that regional variations exist in India, and these need to be explored further in a systematic manner.

Abdominal discomfort with defecation occurred in 8.5% of our study population and was significantly associated only in men with loose stools. This association may not be clinically significant as loose stools occurred in a very small proportion of the male subjects (5.7%), and defecation‐associated abdominal pain cannot be extrapolated to imply irritable bowel syndrome.

Noninclusion of lifestyle variables like diet, exercise, and subjective reporting of ineffective bowel clearance in our questionnaire is a limitation of the study.

Furthermore, the purposive sampling may not represent the entire population as our study population's age was skewed to the left (overall median age of 32 years), but we speculate that any healthy noncomplainant population will be skewed in this manner as older people are more likely to have complaints and are hence not included in noncomplainant studies. Another possible limitation of the study is the use of a nonvalidated questionnaire. While all available questionnaires are validated only for disease, our pro forma was designed as a screening tool to obtain a snapshot of a self‐declared healthy individuals’ bowel habits rather than an attempt to categorize disease.

In summary, our study has provided information on the distribution of stool form and its association with gender in a cross section of apparently healthy adult residents of Chennai city. Larger detailed studies across India in noncomplainant populations are needed to confirm regional variations and establish normal stool patterns across India.
